# 

**Published:** 1991-09

**Authors:** 


W1 WE403

V.106 NO.3 1991

C.01--------SEQ: SR0068788

TI: WEST OF ENGLAND MEDICAL

JOURNAL

### WEST OF ENGLAND MEDICAL JOURNAL

Volume 106(iii) September 1991

The Waldegrave Interview
The Bristol Royal Infirmary as a Trust Hospital — the first six months
Private Practice — does it have a place?
Thoughts on the NHS
Job-sharing in General Practice
The Cornwall Helicopter
Recreational deaths from Drowning
Malignant neuro-endocrinoma of the Pancreas
Ultrasound of the Infant Hip
Why I like chest physicians
From our Correspondents
Abstracts of meetings

NATIONAL LIBRARY
FEB 14 1992
OF MEDICINE

FORMERLY BRISTOL MEDICO-CHIRURGICAL JOURNAL

ISSN: 0960 6440 NLM Code B5Q

